# Does Relationship Status Affect the Association Between Comorbidity, Physical Activity and Frailty in Older People? Findings from the Southampton Longitudinal Study of Ageing (SaLSA)

**DOI:** 10.3390/geriatrics11050148

**Published:** 2026-10-19

**Authors:** Faidra Laskou, Leo D. Westbury, Fiona Kirkham-Wilson, Gregorio Bevilacqua, Nicholas R. Fuggle, Pritti Aggarwal, Elaine M. Dennison, Harnish P. Patel

**Affiliations:** 1 Lifecourse Epidemiology Centre, University of Southampton, Southampton SO16 6YD, UK; f.laskou@soton.ac.uk (F.L.); lw@mrc.soton.ac.uk (L.D.W.); f.kirkham-wilson@soton.ac.uk (F.K.-W.); gregorio.bevilacqua@advocatehealth.org (G.B.); nrf@mrc.soton.ac.uk (N.R.F.); emd@mrc.soton.ac.uk (E.M.D.); 2 Living Well Partnership, Southampton SO19 9GH, UK; prittiaggarwal@nhs.net; 3 NIHR Southampton Biomedical Research Centre, University of Southampton and University Hospital Southampton NHS Foundation Trust, Southampton SO16 6YD, UK; 4 School of Biological Sciences, Victoria University of Wellington, Wellington 6012, New Zealand; 5 Academic Geriatric Medicine, University of Southampton, Southampton SO16 6YD, UK

**Keywords:** comorbidity, physical activity, frailty, educational attainment, relationship

## Abstract

**Background**: Comorbidity is common in older adults and is associated with lower physical activity (PA) and greater frailty risk. We assessed whether relationship status affects these relationships, using the Southampton Longitudinal Study of Ageing. **Methods**: 448 community-dwelling older participants (median age of 80 years) were analysed. A questionnaire ascertained relationship status, number of comorbidities, PA (Physical Activity Scale for the Elderly) and frailty components (weight loss, self-reported exhaustion, low PA, low self-reported walking speed, difficulty carrying 10 lb). Associations of number of comorbidities with physical activity and number of frailty components were examined using linear and Poisson regression, respectively. Models were adjusted for age, sex, BMI and smoking status and included an interaction term between number of comorbidities and relationship status. Sex-stratified analyses without interactions were also performed. **Results:** Evidence of interaction effects were weak (*p* > 0.14 for both tests). Among females, higher comorbidity was related to lower PA and a greater number of frailty components: difference in PA (95% CI) and multiplicative increase in number of frailty components (95% CI) per additional comorbidity was −0.10 (−0.17,−0.03) and 1.25 (1.16,1.34) respectively. Effect sizes for PA and number of frailty components were broadly similar among females when stratified according to relationship status. Associations were weaker among males (*p* > 0.27 for all associations). **Conclusions**: Evidence of relationship status modifying these associations was weak. Females with greater comorbidity had lower physical activity and more frailty components. Differences in observed strength of associations between males and females may reflect different comorbidity profiles.

## 1. Introduction

Multimorbidity is the presence of two or more chronic health conditions in an individual. It is an increasing public health concern and imposes a considerable economic burden on health systems and wider society [1]. In a whole population study of over 60 million people in England, the overall prevalence of multimorbidity was 14.8% and increased to 68.2% in those aged 80 years and over [2]. As life expectancy rises, the burden of multimorbidity is projected to increase considerably, with a projected increase of 84% in the number of people with multimorbidity from 2019 to 2049 [3]. Individuals with multimorbidity often experience poorer health outcomes, including higher risks of disability, hospital admission, and mortality, as well as reduced quality of life [4].

Previous systematic reviews have established associations between multimorbidity (or greater comorbidity burden) and both lower physical activity [5] and increased risk of frailty [6] among older adults. Marital status has been suggested as a predictor of lower risk of comorbidity, with effects apparently mediated through lifestyle [7].

Relationship status may influence not only health behaviours, but also health seeking behaviours and outcomes through mechanisms such as social support, caregiving, and shared lifestyle habits [8]. Finally, benefits of being in a relationship may vary by sex, with males traditionally appearing to gain more healthwise from marriage than females, in whom less striking differences are apparent [9].

We therefore examined the associations between number of comorbidities, physical activity, and number of frailty components in older adults, considering whether these relationships differ according to relationship status. We also examined whether associations regarding number of comorbidities with physical activity and frailty differ by sex and educational attainment as these factors may influence the extent to which comorbidity burden is associated with physical functioning in later life. Our study used data from the Southampton Longitudinal Study of Ageing (SaLSA), a population-based cohort of older adults in the United Kingdom.

## 2. Methods

### 2.1. Study Design

The Southampton Longitudinal Study of Ageing (SaLSA) comprises community-dwelling males and females over the age of 75 years in Southampton, UK, who were recruited between 2021 and 2022. The aim of this study was to create a new local cohort of older adults in the post-pandemic era to investigate the effects of lifestyle and ageing on general and musculoskeletal health. Participants were identified from the general practice database, with the only inclusion criterion being their age at the time of recruitment. Eligibility was determined by their primary care physician. Exclusion criteria included individuals with safeguarding issues, mental health problems, impaired mental capacity, dementia, inability to provide consent, learning disabilities, those on end-of-life care plans, those in residential or nursing homes, and those who were bed-bound. Detailed recruitment methods for SaLSA have been previously described [10].

### 2.2. Ascertainment of Participant Information

All eligible participants were sent a postal questionnaire to complete. Information ascertained for this study included self-reported height and weight; medical conditions and medical history; smoking status (categorised into current/ex versus never); physical activity (Physical Activity Scale for the Elderly [PASE]); educational attainment (categorised into university degree/Higher National Diploma/higher professional qualifications versus O Levels/GCSEs/A Levels/vocational training certificates); and relationship status (categorised into married/civil partnership/cohabiting versus single/divorced/separated/widowed).

Participants also self-reported the following information: lost more than 10 pounds (lb) unintentionally in the past year (yes vs. no); number of days per week that they felt that everything they did was an effort or that they could not get going (<1, 1–2, 3–4, >4); self-reported walking speed (fast, fairly brisk, normal speed, stroll at an easy pace, very slow, unable to walk); and difficulty carrying 10 lb (none, some, a lot/unable). This information was then used to derive an adapted frailty phenotype-participants with ≥ 3 of the following were regarded as living with frailty: lost more than 10 lb unintentionally in the past year; self-reported exhaustion in the past week (≥3 days); low physical activity (bottom sex-specific fifth of the distribution); low self-reported walking speed (very slow or unable to walk); or difficulty carrying 10 lb (a lot of trouble or unable). A score for the number of frailty components, ranging from 0 to 5, was then derived from these components and used as the primary frailty variable in analyses.

Comorbidities were ascertained by asking participants whether they were ever diagnosed by a doctor with any of the following conditions: heart attack or angina; stroke or transient ischaemic attack; hypertension; diabetes; asthma, bronchitis, emphysema, or chronic obstructive pulmonary disease (COPD); depression; osteoporosis; anxiety; memory problems or dementia; Parkinson’s disease; osteoarthritis or degenerative joint disease; rheumatoid/inflammatory arthritis; cancer; or high cholesterol. The resulting number of comorbidities reported was used as a marker of morbidity level.

### 2.3. Ethics Approval

Ethics and HRA approval were obtained from South Central Oxford Research Ethics Committee reference 21/SC/0036, project ID 288051. Informed written consent was obtained from all participants.

### 2.4. Statistical Methods

Summary statistics were used to describe participant characteristics. The physical activity score was standardized such that it had a mean of zero and a standard deviation of one to aid interpretation of effect sizes. Associations of number of comorbidities with physical activity and number of frailty components were examined using linear and Poisson regression, respectively. Models were adjusted for age, sex, BMI and smoking status and included an interaction term between number of comorbidities and relationship status. Formal tests of interaction were also performed to assess whether associations of comorbidity burden with physical activity and frailty differed by sex and educational attainment. Comorbidity-by-sex interactions were examined in the overall sample, while comorbidity-by-relationship status and comorbidity-by-educational attainment interactions were examined in both sex-pooled and sex-stratified analyses. Corresponding interaction *p*-values were reported.

Number of comorbidities in relation to physical activity and number of frailty components was also examined without inclusion of interaction terms using sex-stratified linear and Poisson regression respectively. Analyses were adjusted for age, BMI and smoking status. These were selected as adjustment variables because of their highly plausible relationships with both comorbidity burden and the study outcomes, rather than according to their statistical associations in the present dataset.

Poisson regression with robust variance estimation was used to examine associations between number of comorbidities and the number of frailty components, as this outcome was a count variable. Robust variance estimation was used to ensure that the modelling approach was robust to mild departures from the variance assumptions of ordinary Poisson regression. Model adequacy was assessed by examining residuals, including plots of residuals against fitted values, which showed no substantial systematic departures from the modelling assumptions.

Of the 516 SaLSA participants, 4 had missing information on relationship status or educational attainment. Of the remaining 512 participants, 34 had missing information on number of frailty components or physical activity. Of the remaining 478 participants, 30 had missing information on BMI or smoking status. Therefore, the analysis sample comprised the 448 participants with complete data regarding all the variables of interest.

Sensitivity analyses included examining number of comorbidities in relation to number of frailty components using a modified frailty score which did not include the physical activity component, to investigate whether associations with the frailty outcome were robust to the removal of the physical activity component.

To assess potential differences between participants included in the analytical sample and those excluded because of missing data, selected characteristics were compared between these groups. These characteristics included sex, BMI, smoking status and educational attainment, where available. For males and females separately, the number and percentage with each comorbidity used to derive the number of comorbidities were also calculated. Analyses were conducted using Stata, version 17 (StataCorp LLC, College Station, TX, USA); *p* < 0.05 was regarded as statistically significant.

## 3. Results

### 3.1. Participant Characteristics

Table 1 illustrates the participant characteristics of the analysis sample. Median age was around 80 years. Median (lower quartile, upper quartile) physical activity scores were 129 (86, 171) among males and 96 (69, 134) among females; corresponding values for number of comorbidities were 2 (1, 3) among both males and females. The number of participants with frailty (three or more frailty components) was 15 (7%) among males and 32 (14%) among females.

### 3.2. Number of Comorbidities in Relation to Physical Activity and Number of Frailty Components

Table 2 presents the *p*-values from tests of formal interaction effects between number of comorbidities and sociodemographic factors on physical activity and frailty outcomes after adjustment for age, BMI, and smoking status. In the entire analysis sample after additional adjustment for sex, evidence of an interaction effect between number of comorbidities and relationship status was weak for outcomes of physical activity (*p* = 0.842) and number of frailty components (*p* = 0.141). Significant interactions were observed between comorbidity burden and sex for the number of frailty components (*p* = 0.015) and between comorbidity burden and relationship status among females for the number of frailty components (*p* = 0.048). No other interaction effects examined reached statistical significance (*p* > 0.05 for remaining tests).

Associations for number of comorbidities in relation to physical activity and number of frailty components after stratification by sex and without inclusion of interaction terms are presented in Table 3. Among females, higher comorbidity was related to lower physical activity and a greater number of frailty components: difference in physical activity (95% CI) and multiplicative increase in number of frailty components (95% CI) per additional comorbidity was −0.10 (−0.17, −0.03) and 1.25 (1.16, 1.34) respectively. Despite the significant interaction observed between comorbidity burden and relationship status among females for the number of frailty components (*p* = 0.048) described above, effect sizes for physical activity and number of frailty components per additional comorbidity were broadly similar among females when stratified according to relationship status and educational attainment. For example, the multiplicative increase in number of frailty components was 1.39 (1.25, 1.56) for females who were married, in a civil partnership or cohabiting, and 1.20 (1.10, 1.32) for females who were single, divorced, separated or widowed; corresponding estimates for those who had a university degree, Higher National Diploma or higher professional qualification, and those who did not have such qualifications were 1.33 (1.09, 1.62) and 1.25 (1.16, 1.35) respectively. In contrast, associations regarding number of comorbidities in relation to physical activity and number of frailty components appeared weaker among males compared to females, regardless of how the analysis sample was stratified (*p* > 0.27 for all associations). However, as described in the paragraph above, there was only evidence of an interaction between number of comorbidities and sex for number of frailty components (*p* = 0.015), and not for physical activity (*p* = 0.215).

Supplementary Table S1 presents associations for number of comorbidities in relation to the number of frailty components when physical activity was not included as a component in the derivation of the number of frailty components. These associations were similar to those observed in Table 3.

Compared with participants included in the analysis sample, those not included were more likely to be female (68% vs. 53%; *p* = 0.023) and less likely to have ever smoked regularly (31% vs. 50%; *p* = 0.004), whereas BMI and educational attainment did not differ significantly between groups (both *p* > 0.05) (Supplementary Table S2). Supplementary Table S3 reports the prevalence of each comorbidity according to sex.

## 4. Discussion

In this UK study comprising community-dwelling older males and females, evidence that relationship status modifies associations of comorbidity burden with physical activity and number of frailty components was weak. Greater comorbidity burden was related to lower physical activity and higher numbers of frailty components among females, with similar associations when stratified according to relationship status. Although a significant interaction was observed between comorbidity burden and relationship status among females for the number of frailty components (*p* = 0.048), this borderline significant association must be interpreted in the context of the number of interaction tests performed, and the lack of evidence of such an interaction in the pooled sample. Although associations of number of comorbidities with physical activity and number of frailty components appeared weaker among males compared to females, there was only evidence of an interaction between number of comorbidities and sex for number of frailty components (*p* = 0.015), and not for physical activity (*p* = 0.215). Our sample size was relatively small, but these observations warrant study and replications in other, larger samples.

Previous studies have examined sex-specific associations between multimorbidity (and comorbidity burden) and physical activity, and to a lesser extent between multimorbidity and frailty [5,6]. Most recently, a systematic review of observational studies examining the association between physical activity and multimorbidity in older adults reported an inverse association, based on a meta-analysis of more than 77,000 participants [5]. Of the 15 studies included in this systematic review, nine found an inverse association in both sexes, three found it only in males, and three found no association. In general, relationships were stronger in males, in contrast to our own observations [5]. Over the past few years, a small number of studies examined this association, providing further conflicting evidence [11,12]. Higher chronic disease burden was significantly associated with lower physical activity levels in a US longitudinal study, with no sex differences observed [11], while a Brazilian study found higher levels of physical activity among females with multimorbidity, compared to their peers without multimorbidity [12]. Sampling and methodological issues may underlie these differences.

The potential benefits of physical activity may also extend beyond physical function and frailty to other aspects of healthy ageing, including cognitive health. Recent evidence from older adults has shown that higher levels of leisure-time physical activity were associated with better memory and processing speed, with these associations also varying according to oral microbiome diversity [13]. These findings further support the broader relevance of maintaining physical activity in later life.

In a 2019 systematic review that considered the association of multimorbidity with frailty using eight cross-sectional studies, multimorbidity was associated with frailty (odds ratio (95% CI): 2.27 (1.97–2.62)) [6]. However, all these eight studies pooled males and females, potentially masking sex-differences. Multimorbidity, frailty and decreased physical activity are reported to be more prevalent in females than males [14,15,16], who also enjoy longer life expectancy [17], and important social and biological differences exist between sexes.

We observed stronger associations among community-dwelling older females between higher levels of comorbidity, lower physical activity, and increased number of frailty components, even though tests for an interaction between number of comorbidities and sex were only statistically significant for number of frailty components and not for physical activity. Although these observations need testing in other, larger samples, there are many reasons for this observation, aside from biological differences. For example, the comorbidities described in males and females were different in our sample, with 32% of females and 15% of males reporting a past medical history of osteoarthritis or degenerative joint disease, with hypertension also being more common in females than males (55% vs. 48%), while more males than females reported a past history of cancer (24% vs. 16%). Patterns of physical activity also differed slightly between males and females, with 18% of males and 13% of females reporting regular participation in light or moderate sporting activity regularly, and 10% reporting participation in strenuous sporting activity in both groups. Of course, we cannot tell direction of association, as individuals with poorer physical reserves may be less physically active. The weaker associations observed between comorbidity, physical activity and frailty in males is at first slightly surprising. However, this may reflect an apparent selection bias operating in the cohort, especially among males. For example, a higher proportion of males who participated were in relationships compared to the females who were enrolled, and we observed higher levels of physical activity in males versus females in our sample.

Selection into the study may also have differed according to relationship status and other participant characteristics, potentially influencing the associations observed. However, the extent and direction of any such selection cannot be evaluated using the available data, and the characteristics of non-participants in this respect are unknown. Furthermore, although associations between comorbidity burden and the outcomes varied across some relationship status categories, with slightly smaller effect sizes for number of comorbidities in relation to number of frailty components among females who were single, divorced, separated or widowed compared to those who were not, the present study did not provide clear evidence that relationship status modified these associations. These findings should be interpreted cautiously given the relatively small sample sizes, especially within the stratified analyses.

There are of course several other limitations to this study. All data were collected through self-reported questionnaires, which may introduce recall bias. We adopted the most commonly recommended definition of comorbidity burden but there is no consensus on which chronic diseases should be considered when defining comorbidity burden and multimorbidity [18]. Similarly, the availability of different ways of assessing physical activity presents challenges in drawing direct comparisons across studies employing different methodologies. We have used PASE, which has been validated for use in ageing research and applied across diverse clinical and geographical settings [19]. The exclusion criteria for SaLSA (individuals with dementia or impaired mental capacity, those who were bed-bound, and those living in residential or nursing homes) may have resulted in a relatively healthy study population. Consequently, the findings may be less generalisable to more vulnerable older adults with greater levels of frailty, dependency, or cognitive impairment. Another limitation is that relationship status was dichotomised as married/civil partnership/cohabiting versus single/divorced/separated/widowed, which may have obscured important differences within the latter group in social support, bereavement, lifestyle and living arrangements, particularly in this older population. Individuals with the same relationship status may differ substantially in the amount and quality of social support and in their caregiving circumstances. Therefore, relationship status should not be interpreted as a direct measure of these constructs, which were not assessed. In addition, observed differences in the strength of associations between males and females may partly reflect sex differences in the composition of comorbidity patterns rather than sex itself. Our measure for comorbidity burden was based on a simple count in which all conditions contributed equally, despite conditions having potentially differing relationships with physical activity and frailty. As analyses accounting for specific disease profiles were not undertaken due to the small sample sizes, especially for the stratified analyses, these possibilities could not be distinguished. Similarly, due to the small sample sizes, we were unable to explore whether the associations examined in this article differed according to the specific medical conditions present.

In conclusion, associations between comorbidity burden, physical activity and frailty were broadly similar regardless of relationship status in a sample of older community-dwelling adults. Although associations of number of comorbidities with physical activity and number of frailty components appeared weaker among males compared to females, there was only evidence of an interaction between number of comorbidities and sex for number of frailty components (*p* = 0.015), and not for physical activity (*p* = 0.215). We hypothesise that this may reflect a different comorbidity pattern in the two sexes, possibly coupled with a healthy cohort bias. Further work in larger cohorts drawn from other populations is now warranted.

## Figures and Tables

**Table 1:** Characteristics of the analysis sample.

Participant Characteristic	Males (*n* = 211)	Females (*n* = 237)
Age (years)	79 (77, 83)	80 (77, 84)
BMI (kg/m^2^)	26.0 (3.4)	26.5 (5.1)
Ever smoked regularly	132 (63%)	94 (40%)
Physical activity score (PASE)	129 (86, 171)	96 (69, 134)
Married/cohabiting/civil partnership	148 (70%)	91 (38%)
Education (university degree/HND/higher professional qualifications)	51 (24%)	36 (15%)
Number of comorbidities *	2 (1, 3)	2 (1, 3)
***Frailty components***		
Lost more than 10 pounds in weight unintentionally in past year	21 (10%)	16 (7%)
Self-reported exhaustion in the past week (≥3 days)	39 (18%)	63 (27%)
Low physical activity (bottom sex-specific fifth)	42 (20%)	46 (19%)
Self-reported walking speed (very slow/unable to walk)	34 (16%)	54 (23%)
Carrying 10 pounds (a lot of trouble/unable)	10 (5%)	46 (19%)
***Frailty status***		
Frailty (three or more frailty components)	15 (7%)	32 (14%)
Number of frailty components		
0	127 (60%)	122 (51%)
1	44 (21%)	52 (22%)
2	25 (12%)	31 (13%)
3	9 (4%)	18 (8%)
4	5 (2%)	13 (5%)
5	1 (0%)	1 (0%)
*PASE: Physical Activity Scale for the Elderly*		
*HND: Higher National Diploma*		
*BMI was calculated from self-reported height and weight*		

* Comorbidities were ascertained by asking participants whether they were ever diagnosed by a doctor with any of the following conditions: heart attack or angina; stroke or transient ischaemic attack; hypertension; diabetes; asthma, bronchitis, emphysema, or chronic obstructive pulmonary disease (COPD); depression; osteoporosis; anxiety; memory problems or dementia; Parkinson’s disease; osteoarthritis or degenerative joint disease; rheumatoid/inflammatory arthritis; cancer; or high cholesterol.

**Table 2:** Interaction effects between number of comorbidities and sociodemographic factors on physical activity and number of frailty components after adjustment for age, BMI, and smoking status.

Outcome	Sample	Interaction Term	*p*-Value
Physical activity	Entire sample	Number of comorbidities × relationship status	0.842
Number of frailty components	Entire sample	Number of comorbidities × relationship status	0.141
Physical activity	Males	Number of comorbidities × relationship status	0.269
Number of frailty components	Males	Number of comorbidities × relationship status	0.176
Physical activity	Females	Number of comorbidities × relationship status	0.910
Number of frailty components	Females	Number of comorbidities × relationship status	0.048
Physical activity	Entire sample	Number of comorbidities × educational attainment	0.549
Number of frailty components	Entire sample	Number of comorbidities × educational attainment	0.948
Physical activity	Males	Number of comorbidities × educational attainment	0.488
Number of frailty components	Males	Number of comorbidities × educational attainment	0.270
Physical activity	Females	Number of comorbidities × educational attainment	0.780
Number of frailty components	Females	Number of comorbidities × educational attainment	0.879
Physical activity	Entire sample	Number of comorbidities × sex	0.215
Number of frailty components	Entire sample	Number of comorbidities × sex	0.015

*p*-values are for interaction terms between number of comorbidities and the specified sociodemographic characteristic. Analyses were adjusted for age, BMI, and smoking status; analyses performed among the entire sample also include sex as a main effect. Comorbidities were ascertained by asking participants whether they were ever diagnosed by a doctor with any of the following conditions: heart attack or angina; stroke or transient ischaemic attack; hypertension; diabetes; asthma, bronchitis, emphysema, or chronic obstructive pulmonary disease (COPD); depression; osteoporosis; anxiety; memory problems or dementia; Parkinson’s disease; osteoarthritis or degenerative joint disease; rheumatoid/inflammatory arthritis; cancer; or high cholesterol.

**Table 3:** Number of comorbidities in relation to physical activity and number of frailty components, stratified according to various groups.

Outcome	Analysis Sample	Males		Females	
		Estimate (95% CI)	*p*-Value	Estimate (95% CI)	*p*-Value
Physical activity (standard deviation difference in physical activity per additional comorbidity)	Entire sex-specific sample	−0.03 (−0.14, 0.07)	0.548	−0.10 (−0.17, −0.03)	0.004
	Married, civil partnership, cohabiting	−0.06 (−0.19, 0.06)	0.325	−0.09 (−0.19, 0.02)	0.095
	Single, divorced, separated, widowed	0.06 (−0.12, 0.25)	0.489	−0.11 (−0.20, −0.03)	0.012
	University degree, Higher National Diploma, higher professional qualification	0.05 (−0.21, 0.32)	0.681	−0.14 (−0.34, 0.07)	0.180
	Does not have university degree, Higher National Diploma or higher professional qualification	−0.06 (−0.17, 0.05)	0.300	−0.10 (−0.17, −0.02)	0.009
Number of frailty components (multiplicative increase in number of frailty components per additional comorbidity)	Entire sex-specific sample	1.04 (0.90, 1.21)	0.551	1.25 (1.16, 1.34)	<0.001
	Married, civil partnership, cohabiting	1.12 (0.92, 1.35)	0.272	1.39 (1.25, 1.56)	<0.001
	Single, divorced, separated, widowed	0.93 (0.75, 1.15)	0.490	1.20 (1.10, 1.32)	<0.001
	University degree, Higher National Diploma, higher professional qualification	0.83 (0.55, 1.27)	0.396	1.33 (1.09, 1.62)	0.004
	Does not have university degree, Higher National Diploma or higher professional qualification	1.08 (0.94, 1.25)	0.291	1.25 (1.16, 1.35)	<0.001

All models were adjusted for age, BMI and smoking status (ever vs. never); physical activity (z-score) was examined using linear regression. Number of frailty components was examined using Poisson regression with robust variance estimation; an estimate of 1.2 corresponds to a 20% increase in the number of components; an estimate of 0.8 corresponds to a 20% decrease. Comorbidities were ascertained by asking participants whether they were ever diagnosed by a doctor with any of the following conditions: heart attack or angina; stroke or transient ischaemic attack; hypertension; diabetes; asthma, bronchitis, emphysema, or chronic obstructive pulmonary disease (COPD); depression; osteoporosis; anxiety; memory problems or dementia; Parkinson’s disease; osteoarthritis or degenerative joint disease; rheumatoid/inflammatory arthritis; cancer; or high cholesterol.

## Data Availability

Data relating to this study cannot be shared due to consent restrictions. For researchers interested in using data from the Southampton Longitudinal Study of Ageing for research purposes, please contact HPP or FL.

## Supplementary Materials

The following supporting information can be downloaded at: https://www.mdpi.com/article/10.3390/geriatrics11050148/s1, Supplementary Table S1: Number of comorbidities in relation to number of frailty components (excluding physical activity), stratified according to various groups; Supplementary Table S2: Descriptive statistics for participants, depending on whether or not they were included in the analysis sample; Supplementary Table S3: Number and percentage with each comorbidity, stratified by sex.
