# Supplementary material for: Does Relationship Status Affect the Association Between Comorbidity, Physical Activity and Frailty in Older People? Findings from the Southampton Longitudinal Study of Ageing (SaLSA)
Source: Geriatrics (Basel). 2026 Oct 19;11(5):148. doi: 10.3390/geriatrics11050148 (PMC13704052; doi:10.3390/geriatrics11050148)
Supplement: Supplementary file 1 [file geriatrics-11-00148-s001.zip › geriatrics-4527376-supplementary.pdf]

Does relationship status affect the relationship between comorbidity, physical activity and frailty in older people? Findings from the Southampton Longitudinal Study of Ageing (SaLSA)

Faidra Laskou [1*], Leo D Westbury [1*], Fiona Kirkham-Wilson [1], Gregorio Bevilacqua [1], Nicholas R Fuggle [1], Pritti Aggarwal [2], Elaine M Dennison [1,3,4] and Harnish P Patel [1,3,5 +]

Supplementary tables

Supplementary Table S1: Number of comorbidities in relation to number of frailty components (excluding physical activity), stratified according to various groups

Outcome	Analysis sample	Males		Females	
		Estimate (95% CI)	P-value	Estimate (95% CI)	P-value
Number of frailty components (multiplicative increase in number of frailty components per additional comorbidity)	Entire sex-specific sample	1.08 (0.92,1.27)	0.344	1.28 (1.18,1.38)	<0.001
	Married, civil partnership, cohabiting	1.17 (0.95,1.45)	0.136	1.42 (1.25,1.62)	<0.001
	Single, divorced, separated, widowed	0.94 (0.74,1.20)	0.621	1.23 (1.12,1.35)	<0.001
	University degree, Higher National Diploma, higher professional qualification	0.87 (0.54,1.40)	0.562	1.50 (1.23,1.83)	<0.001
	Does not have university degree, Higher National Diploma or higher professional qualification	1.11 (0.95,1.31)	0.186	1.28 (1.18,1.38)	<0.001

All models were adjusted for age, BMI and smoking status (ever vs never)
Number of frailty components was examined using Poisson regression with robust variance estimation; an estimate of 1.2 corresponds to a 20% increase in the number of components; an estimate of 0.8 corresponds to a 20% decrease.
Comorbidities were ascertained by asking participants whether they were ever diagnosed by a doctor with any of the following conditions: heart attack or angina; stroke or transient ischaemic attack; hypertension; diabetes; asthma, bronchitis, emphysema, or chronic obstructive pulmonary disease (COPD); depression; osteoporosis; anxiety; memory problems or dementia; Parkinson's disease; osteoarthritis or degenerative joint disease; rheumatoid/inflammatory arthritis; cancer; or high cholesterol

Supplementary Table S2: Descriptive statistics for participants, depending on whether or not they were included in the analysis sample

Participant characteristic	Not included (n=68)	Included (n=448)	P-value
Female sex	46 (68%)	237 (53%)	0.023
BMI (kg/m^2)	25.1 (4.4)	26.3 (4.4)	0.111
Ever smoked regularly	20 (31%)	226 (50%)	0.004
University degree / HND / higher professional qualifications)	11 (16%)	87 (19%)	0.525

HND: Higher National Diploma

Supplementary Table S3: Number and percentage with each comorbidity, stratified by sex

Condition	Men (n=211)	Women (n=237)
Heart attack or angina	24 (11%)	12 (5%)
Stroke or transient ischaemic attack	18 (9%)	17 (7%)
High blood pressure	102 (48%)	131 (55%)
Type 1 or type 2 diabetes	35 (17%)	26 (11%)
Asthma, bronchitis, emphysema, or COPD	36 (17%)	43 (18%)
Depression	10 (5%)	29 (12%)
Osteoporosis	5 (2%)	43 (18%)
Anxiety	4 (2%)	21 (9%)
Memory problems or dementia	4 (2%)	2 (1%)
Parkinson's disease	3 (1%)	2 (1%)
Osteoarthritis or degenerative joint disease	32 (15%)	75 (32%)
Rheumatoid/inflammatory arthritis	21 (10%)	44 (19%)
Cancer	50 (24%)	38 (16%)
High cholesterol	73 (35%)	78 (33%)

COPD: chronic obstructive pulmonary disease
